# A novel Schmallenberg virus subunit vaccine candidate protects IFNAR^-/-^ mice against virulent SBV challenge

**DOI:** 10.1038/s41598-020-73424-2

**Published:** 2020-11-23

**Authors:** Hani Boshra, Gema Lorenzo, Diego Charro, Sandra Moreno, Gabriel Soares Guerra, Isbene Sanchez, Joseba M. Garrido, Marivi Geijo, Alejandro Brun, Nicola G. A. Abrescia

**Affiliations:** 1 Center for Cooperative Research in Biosciences (CIC bioGUNE), Basque Research and Technology Alliance (BRTA), Bizkaia Technology Park, 48160 Derio, Spain; 2 Animal Health Research Center (INIA-CISA), 28130 Valdeolmos, Madrid, Spain; 3 Vacunek SL, Bizkaia Technology Park, 48160 Derio, Spain; 4 Animal Health Department, NEIKER-Basque Institute for Agricultural Research and Development, Derio, Bizkaia Spain; 5 Basque Foundation for Science, IKERBASQUE, 48013 Bilbao, Spain; 6 Centro de Investigación Biomédica en Red de Enfermedades Hepáticas y Digestivas (CIBERehd), Instituto de Salud Carlos III, Madrid, Spain; 7 Present Address: Department of Pathology, Fundamental and Applied Research for Animals & Health (FARAH), Faculty of Veterinary Medicine, University of Liège, Bât B43, avenue de Cureghem 6, 4000 Liège, Belgium

**Keywords:** Vaccines, Adjuvants, Protein vaccines, Infection, Viral infection, Virology, Viral pathogenesis

## Abstract

Schmallenberg virus (SBV), an arthropod-transmitted pathogenic bunyavirus, continues to be a threat to the European livestock industry, causing morbidity and mortality among young ruminant livestock. Here, we describe a novel SBV subunit vaccine, based on bacterially expressed SBV nucleoprotein (SBV-N) administered with a veterinary-grade Saponin adjuvant. When assayed in an IFNAR^-/-^ mouse model, SBV-N with Saponin induced strong non-neutralizing broadly virus-reactive antibodies, decreased clinical signs, as well as significantly reduced viremia. Vaccination assays also suggest that this level of immune protection is cell mediated, as evidenced by the lack of neutralizing antibodies*,* as well as interferon-γ secretion observed in vitro. Therefore, based on these results, bacterially expressed SBV-N, co-administered with veterinary-grade Saponin adjuvant may serve as a promising economical alternative to current SBV vaccines, and warrant further evaluation in large ruminant animal models. Moreover, we propose that this strategy may be applicable to other bunyaviruses.

## Introduction

Schmallenberg virus (SBV) is an orthobunyavirus of the family *Peribunyaviridae*, of the newly established order *Bunyavirales*^1^. A negative stranded, tri-segmented RNA virus, SBV was first discovered in 2011, in what would ultimately become a Europe-wide epidemic^2–4^. While symptoms of SBV infection are mild in adult ruminants, SBV has been associated with congenital deformities and stillbirths in newborn ruminants^5^. SBV is an arbovirus, transmitted by biting midges (i.e. Culicoides)^6–8^, and has been found to infect both domestic and wild ruminant species such as sheep, goat, cattle, deer and bison^9–11^. With the initial SBV outbreak lasting from 2011 to 2012, reported cases of SBV infection decreased until 2016, when SBV once again re-emerged across Europe, and lasted well into 2017^12,13^. It was proposed that the first outbreak created a level of herd-immunity that eventually decreased as the number of naïve ruminants increased and, in turn, this increase ultimately led to the latest outbreak^14^. Another factor which may have played a role in the most recent outbreak is the low level of vaccination administered to young and naïve livestock due to the doubts casted on the cost-benefit of current SBV vaccines^15^. While the inactivated SBV vaccines have been available as early as 2013, these vaccines were not commercially successful, as suggested by the fact that by the second SBV outbreak in 2016, two vaccine producers had temporarily halted SBV vaccine production due to their growing financial losses^16,17^. Another example of the economic limitations of current SBV vaccination was demonstrated by a survey conducted by Stokes et al.^16^, where only 20% of UK livestock producers reported previously using SBV vaccines, but 80% would consider doing so if SBV vaccines were priced at 1 (British) pound per dose.

Aside from inactivated vaccines, other potential vaccine candidates against SBV infection have been described. Previously, using cDNA vaccines encoded by the SBV genome, we identified two potential vaccine targets; one based on the nucleoprotein (N) encoded on the S-segment of the genome, and another cDNA segment corresponding to the M-segment of the genome, putatively encoding a portion of the glycoprotein C (Gc) (aa. 678–947)^18^. In both cases, the immune protection conferred to IFNAR^-/-^ mice was not-related with the induction of in vitro neutralizing antibodies. Furthermore, both vaccine targets elicited potent CD8^+^ T-cell proliferation upon in vitro re-stimulation with inactivated SBV virus, suggesting that the protective immunity conferred to mice using these DNA constructs was primarily cell-mediated. Concomitantly, it was shown that another amino-terminal portion of SBV Gc (aa. 467–701) was found to confer immune protection via neutralizing antibodies, when expressed as a fusion protein to a fragment of Gc from the related Akabane virus^19^. Subsequent studies also showed that this SBV Gc fragment could also confer protection when recombinantly expressed using a modified vaccinia Ankara (MVA) virus vector^20^.

While our previous work has detailed vaccine strategies against SBV through DNA vaccination^18^, and work by others the recombinant mammalian cell expression of fused viral proteins^19,20^, the idea of a simpler and more cost-effective SBV subunit vaccine has yet to be explored. It is with these financial constraints in mind that we propose to generate a more economical SBV vaccine, based on our previous findings that SBV nucleoprotein does have immunoprotective properties based on a potent CD8^+^-T-cell response.

Previous structural work has also shown that SBV-N can be recombinantly expressed in bacteria^21^; in particular, bacterially-expressed SBV-N retains the ability to multimerize, bind to RNA and DNA, as well as cross-react to serum from SBV-infected animals^21–24^. We thus hypothesized that a recombinant SBV nucleoprotein (SBV-N) can elicit a similar response if administered as a subunit vaccine; and that this response can be further augmented if SBV-N is administered with Saponin, an adjuvant that can stimulate a potent cell-mediated immune response^25^.

Here, we show that, when administered with Saponin, recombinantly expressed SBV-N can augment a cell-mediated immunological response against virulent SBV infection. The ability of these vaccines to protect against virulent SBV has been evaluated using interferon α/β (IFNAR) knockout mice, a small-animal model that has previously been shown to validate SBV vaccine candidates. Correlates of protection can be observed through increased broadly virus-reactive titers, decreased clinical signs and near undetectable levels of viremia.

These results suggest that a more cost-efficient subunit vaccine can be generated, which would be of practical interest to livestock producers.

## Results

### Generation of SBV nucleoprotein-specific antibodies following vaccination

**Figure 1:**
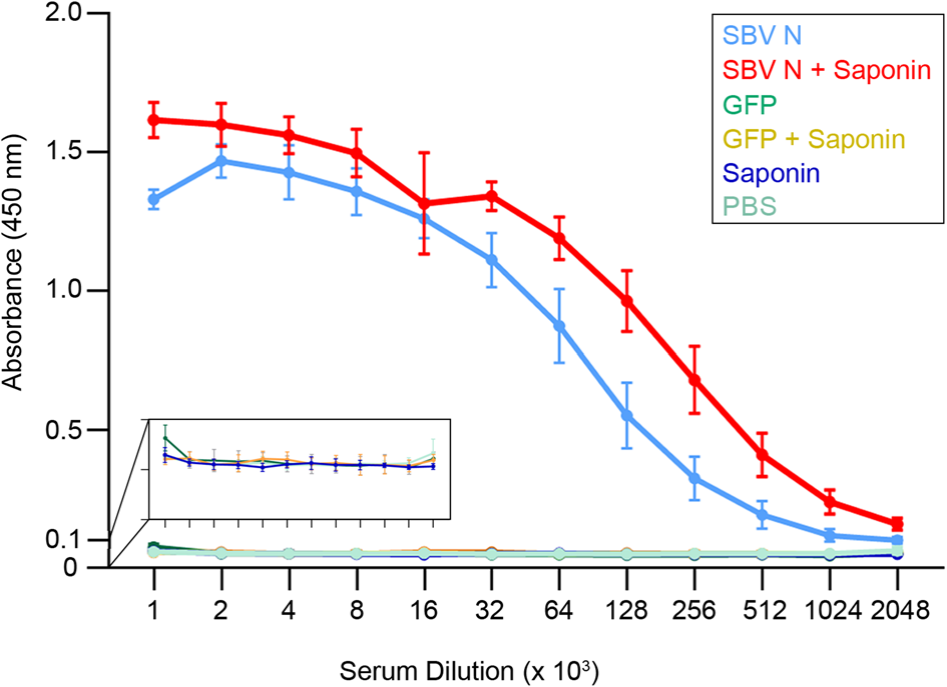
ELISA of sera taken from IFNAR^-/-^ A129 mice following two vaccinations. Sera from all individual mice were tested, starting at a dilution of 1:1 × 10^3^, and assayed to a dilution of 1:2048 × 10^3^. Each point indicates the mean value of each group at 450 nm, as detected using TMB substrate. The one-sided error bars at each point represent the standard deviation. Each of the diluition shows statistical significance as determined using a one-way ANOVA test.

Adult IFNAR^-/-^ mice were inoculated with subunit vaccine candidates (SBV N and SBV N + Saponin) and corresponding controls (GFP, GFP + Saponin, Saponin, PBS) at two week intervals (see “Materials and methods” section). One week following the second set of vaccinations, serum was collected from the six groups of IFNAR^-/-^ mice, and broadly virus-reactive IgG was measured using ELISA. Both SBV-N and SBV-N + Saponin groups had higher broadly virus-reactive antibody titers relative to the other groups with the one-way ANOVA test (see “Materials and methods” section) showing statistical significance among the means across the groups along the twelve dilutions (Fig. 1). When the SBV-N and for the SBV-N + Saponin groups were pairwise compared with the negative control (PBS) group at all dilutions using the t-test, the calculated p-values were statistically significant in each case. Also, when pairwise analysis was performed between the SBV-N and the SBV-N + Saponin groups, SBV-N + Saponin showed statistically greater titers up to dilution 1:256 × 10^3^ (Fig. S1). The results of this pairwise comparison are presented as a sigmoidal curve.

Further, isotyping of the antibodies against SBV-N and SBV-N + Saponin demonstrates that in both cases the IgG1 is the main subclass immunoglobulin responsible for the response (Fig. S2). When IgG isotypes were analyzed from each individual mice from the SBV-N (Fig. S2a) and the SBV-N + Saponin group (Fig. S2b), IgG1 was the predominant IgG isotype in each mouse in both groups. In the SBV-N group, the IgG1/IgG2a ratios varied from 8:1 (Fig. S2a, panel 1) to 1.2:1 (Fig. S2a, panel 6). A similar range was observed in the SBV-N + Saponin group, with IgG1/IgG2a ratios varying from 7:1 (Fig. S2b, panel 3) to 1.1:1 (Fig. S2b, panel 5).

### Virus neutralizing titers of vaccinated IFNAR^-/-^ mice

In order to assess the degree of humoral immunity induced by the different vaccine candidates, the above mentioned sera were also titered for broadly virus-reactive neutralizing antibodies. With dilutions starting at 1:10, no serum from any experimental group exhibited any detectable levels of neutralizing titers (*data not shown*).

### Vaccine efficacy assessment in mice

**Figure 2:**
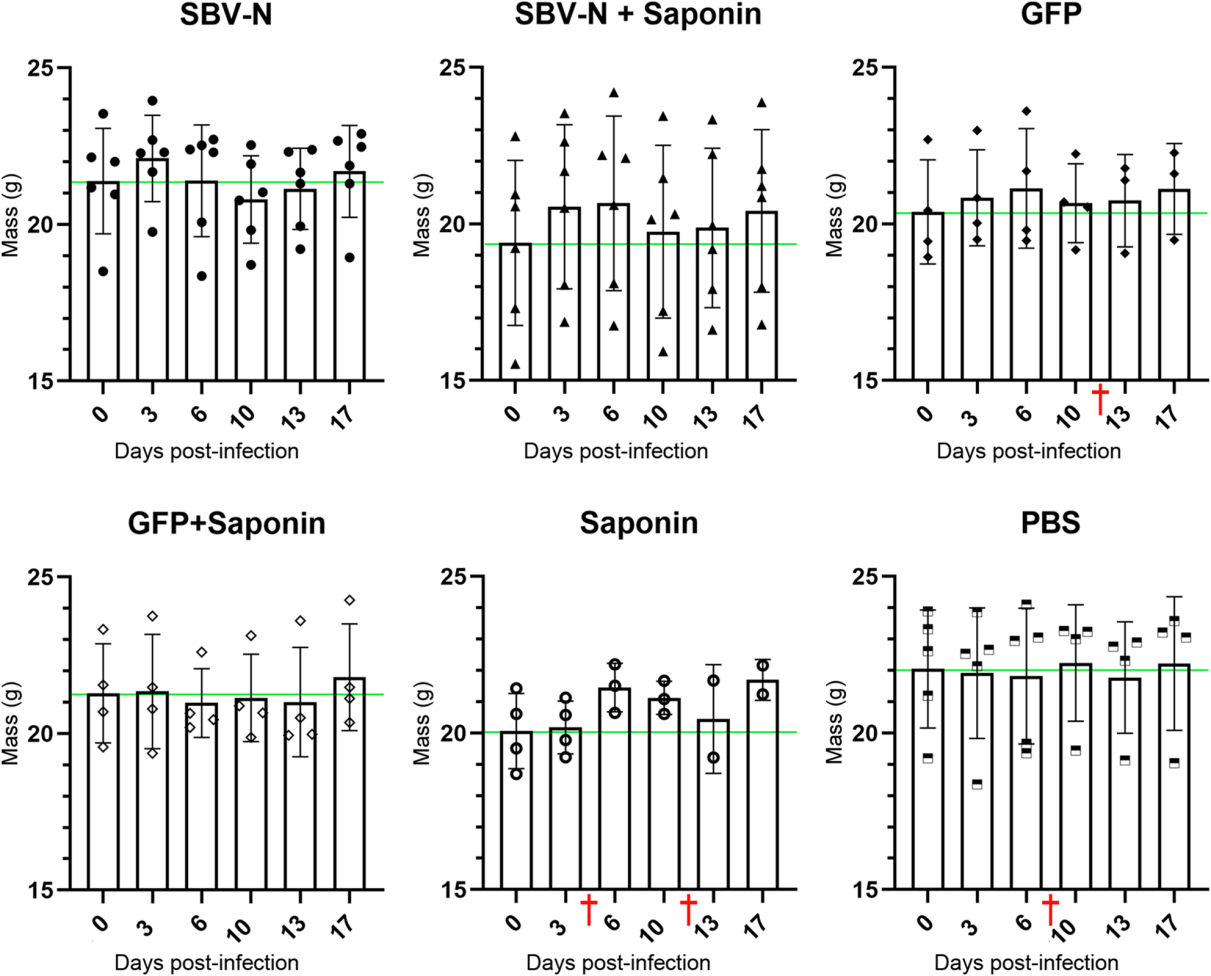
Dot plot measuring the change in weight of all six vaccinated groups following challenge with virulent SBV. Each vaccinated mouse was weighed at 3, 6, 10, 13 and 17 days post-infection (dpi). The histograms show the mean weight of each group with the error bar as the standard deviation from the mean and symbols (black circle, triangle etc.) the individual weight measurements within each group. The green horizontal line denotes the average weight of each group prior to SBV challenge (day 0). Although no statistical significance was found in weight changes after vaccination in each group, three out of the four control groups experienced the death of at least one mouse. Red crosses (†) denote the death of a mouse at a given timepoint.

IFNAR^-/-^ mice were vaccinated twice with either recombinant SBV-N or GFP, with or without Saponin. Two weeks after the final vaccination, the mice were challenged with virulent SBV, administered intraperitoneally (see “Materials and methods” section). The mice were then monitored over the course of 18 days, with each individual mouse being weighed at 0, 3, 6, 10, 13 and 17 dpi. It should be noted that mortality was observed in the GFP, Saponin and PBS groups (denoted by a red cross in Fig. 2). In the Saponin group, two mice died; one on Day 4 and one on Day 13. In the GFP group, one mouse died on Day 10; while in the PBS group, one mouse was found dead on Day 6. The individual weights of each mouse were plotted throughout the period following the viral challenge, with the average weight and corresponding standard deviations of each group indicated (Fig. 2). None of the vaccinated groups displayed statistically significant weight loss (i.e. vis-à-vis average body weight prior to challenge) over the course of the experiment. However, it is worth noting that in three out of four control groups (i.e. GFP, Saponin and PBS), at least one mouse per group died during viral challenge (indicated by the red crosses in Fig. 2).

### Quantification of viremia following SBV challenge

**Figure 3:**
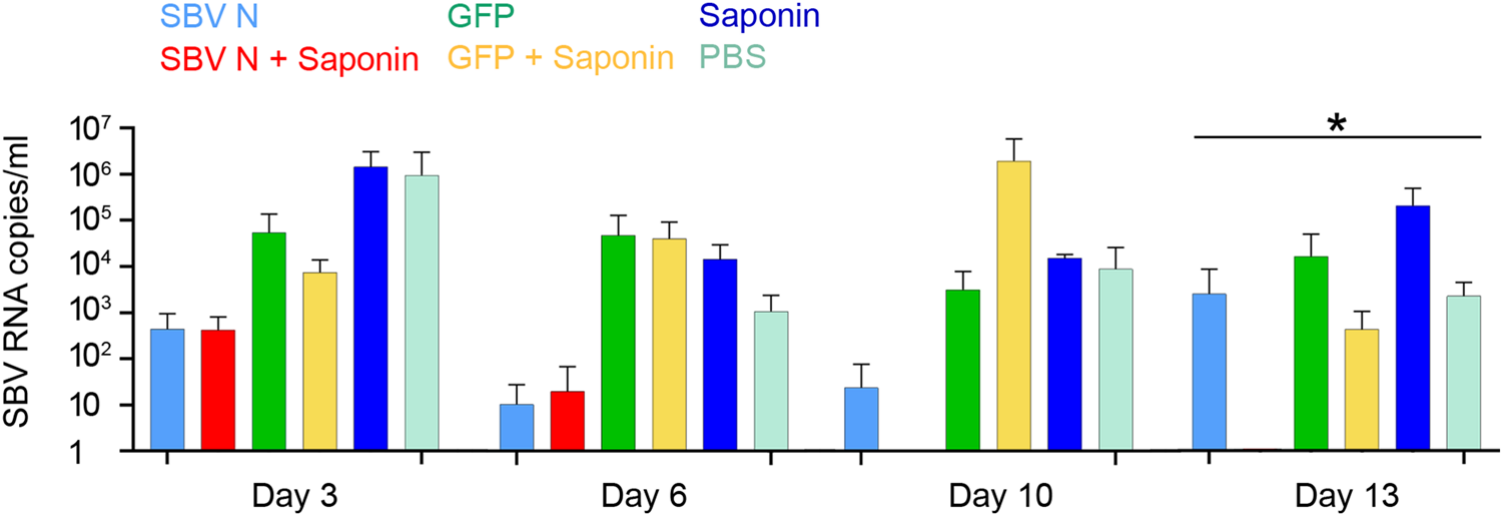
Viremia of A129 mice following viral challenge with virulent SBV. Blood from each vaccinated mouse was taken at 3, 6, 10 and 13 dpi. RNA was extracted from 100 µl of blood, and eluted in the same volume using the extraction technique described in the “Materials and methods” section. Values presented are the number of SBV genome copies/ml. Error bars are presented as the standard deviation from the mean. Asterisk denotes statistical significance, with P < 0.05, as determined by the one-way ANOVA test.

Two weeks after the second vaccination, all mice were subject to SBV challenge using the SBV strain BH619/12, previously characterized to be virulent in IFNAR^-/-^ mice^19^^.^ Approximately 100 µl of blood from each mouse was collected at 3, 6, 10 and 13 dpi. RNA extraction was then performed on each sample, and quantification of genomic SBV was performed using RT-PCR. As seen in Fig. 3, both SBV-N and SBV-N + Saponin had the lowest level of viremia throughout all time points. At Day 3 and 6, both SBV-N and SBV N + Saponin had viral levels of at least an order of magnitude lower, compared to all other vaccinated groups. By Day 10, there were no detectable levels of SBV in any of the 6 mice of the SBV-N + Saponin group, whereas the SBV-N group maintained similar levels relative to Day 3. Interestingly, by Day 13, the SBV-N group displayed a significant increase in viremia, whereas the SBV-N + Saponin group continued to show statistically significant low levels of SBV, with all 6 mice having no detectable levels of SBV RNA. Therefore, the SBV-N + Saponin group demonstrated the lowest degree of SBV-induced viremia throughout the entire experiment.

### Analysis of cellular proliferation upon re-stimulation of splenocytes from vaccinated mice

**Figure 4:**
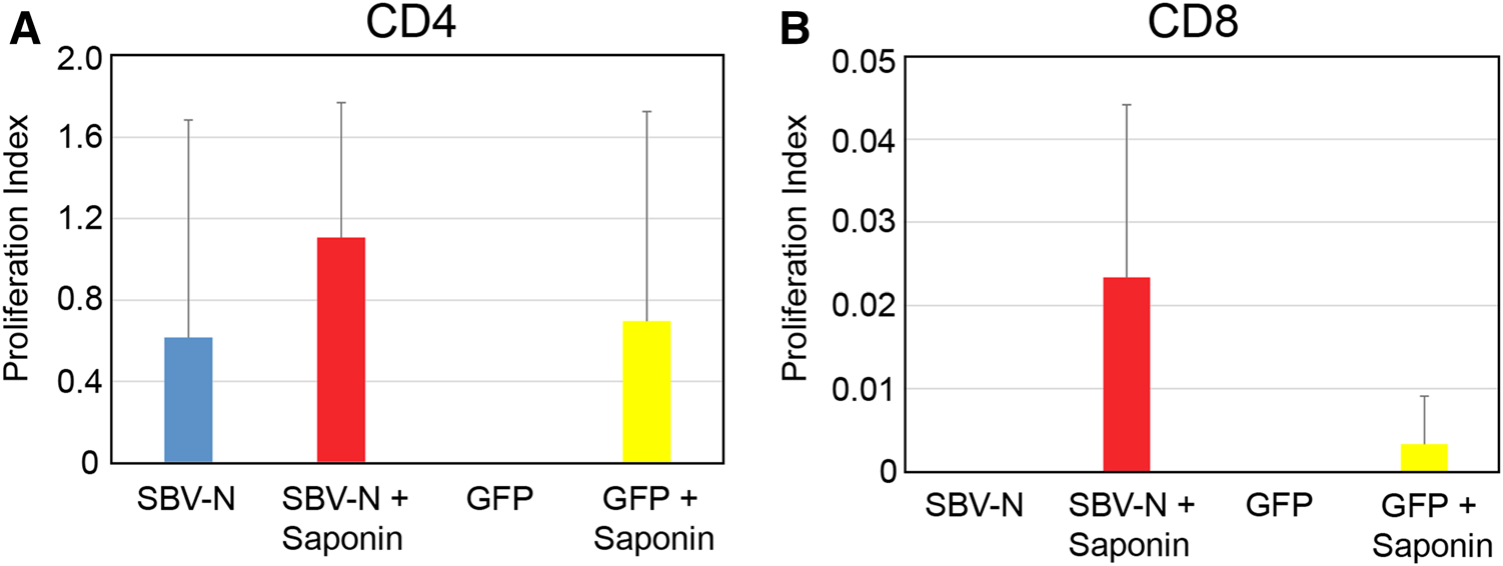
Cellular proliferation of splenocytes from vaccinated IFNAR^-/-^ A129 mice. Splenocytes were extracted from mice vaccinated with one of the following: 1) SBV N; 2) SBV N + Saponin; 3) GFP, or; 4) GFP + Saponin. Splenocytes from each mouse were then incubated with CFSE (see “Materials and methods” section) and stimulated with inactivated SBV for 3 days. The cells were then fixed and incubated with either CD4 (**a**) or CD8-specifc (**b**) antibodies and the proliferation index (average number of divisions of just the responding cells) quantified by flow cytometry. The cell proliferation index is presented as the value of stimulated splenocytes (i.e. in the presence of antigen) minus that of splenocytes under non-stimulating conditions. Error bars are presented as the standard deviation from the mean.

For cell proliferation assays, splenocytes were isolated from the following vaccinated groups: (1) SBV-N; (2) SBV-N + Saponin; (3) GFP, and; (4) GFP + Saponin. The purified splenocytes were then stimulated with heat-inactivated, and enriched SBV. Carboxyfluorescein succinimidyl ester (CFSE) was added to enable for the measurement of cell-proliferation (see “Materials and methods” section). After three days of incubation, the treated splenocytes were incubated with anti-CD4 and CD8 fluorescently-conjugated antibodies. The SBV-N + Saponin group elicited the highest levels of CD8 + T-cell proliferation while in the case of CD4 + T-cells both the SBV-N and SBV-N + Saponin groups demonstrated increased cellular proliferation (relative to the GFP group, which served as the negative control) (Fig. 4); however, none of the three reaching statistical significance (likely due to the relatively small sample size n = 3).

### Detection of interferon-γ secretion in SBV-stimulated splenocytes

**Figure 5:**
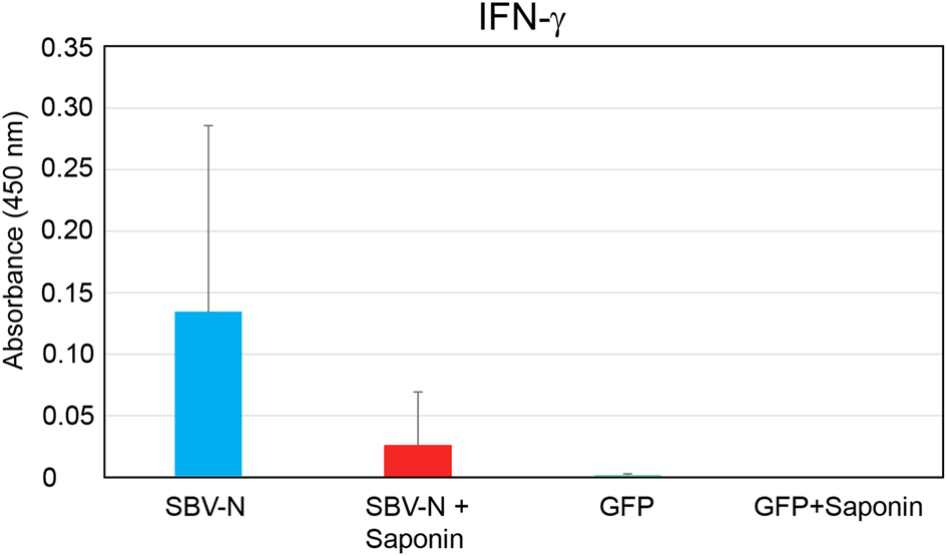
Secreted IFN-γ from splenocytes of vaccinated IFNAR^-/-^ A129 mice. Splenocytes were extracted from mice vaccinated with one of the following: 1) SBV N; 2) SBV N + Saponin; 3) GFP, or; 4) GFP + Saponin. The cells were then isolated and stimulated ex vivo using inactivated SBV. Detection of secreted IFN-γ was measured using ELISA and measured using a spectrophotometer at 450 nm. Error bars are presented as the standard deviation from the mean.

Using conditions similar to those used for ex-vivo CD4 + /CD8 + T-cell proliferation assays, the supernatants from SBV-stimulated splenocytes from vaccinated were assayed for secreted interferon-γ. Both SBV-N groups secreted IFN-γ, with the SBV-N group secreting higher levels than the SBV-N + Saponin group (Fig. 5). In the case of GFP, the amount of IFN-γ was nearly undetectable, whereas the GFP + Saponin group had levels below the sensitivity of the IFN-γ ELISA.

## Discussion

In our previous study of DNA vaccination against SBV infection, we found that two different cDNAs from the SBV genome could protect IFNAR^-/-^ mice from viral challenge^18^. These two components encoded for the nucleoprotein and a putative ectodomain of glycoprotein Gc (aa. 678–947). Similarly, other studies have shown that the N-terminal portion of SBV-Gc (aa. 467–701) when recombinantly expressed in mammalian cells, could also protect IFNAR^-/-^ mice from the virulent viral challenge^19^. Therefore, we decided to evaluate the immunoprotective properties of bacterially-expressed SBV-N using a similar IFNAR^-/-^ mouse model^26^. SBV-N has been previously shown to be highly expressed in bacteria; and contrary to bacterially-expressed glycoproteins, SBV-N has a high degree of solubility, can multimerize, and retain its ability to bind nucleic acids^21,22^. Furthermore, its lack of putative N-linked glycoproteins suggests that most of its biological properties could be retained when recombinantly expressed in bacteria.

While the two aforementioned studies used IFNAR^-/-^ mice as an animal model, one noticeable difference between both experimental designs involved the use of differing strains of SBV. In our previous DNA vaccine studies, vaccinated mice were challenged with SBV strain BH80/11-4, a strain that had been previously shown to induce weight loss in IFNAR^-/-^ mice^19,27^ thus providing a clear clinical sign of virus-induced pathogenesis. However, the work performed by Wernike et al*.* utilized the SBV strain BH619/12 (Ref. 19). While this strain was reported as being able to induce morbidity, including weight loss, in IFNAR^-/-^ mice, it was also described as being partially-lethal in unprotected mice. Therefore, we decided to use this strain to evaluate the subunit vaccine candidates in this study. It is also worth mentioning that, when determining the optimal conditions required for SBV challenge experiments, we found that clinical signs and death were more apparent when the virulent SBV was administered intraperitoneally (compared to the subcutaneous inoculation previously reported), while using slightly more virus, i.e. 2 × 10^5.8^ TCID_50_ *versus* 10^4^ TCID_50_. It is possible that the discrepancies in optimal viral challenge conditions may have been due to the different strain of mice used, with the previous work by Wernike et al*.*^19^ using C57/BL6 IFNAR^-/-^ mice, compared to our use of A129 mice. Nonetheless, in the mouse infection model used here, morbidity and mortality upon BH619/12 challenge were not reliable indicators of vaccine efficacy. Rather, viremia assessment offered a more accurate estimation of the protective capabilities of these vaccines.

We had previously shown that, as a DNA vaccine, SBV-N induced immune protection through cell-mediated immunity^18^; and that CD8 + T-cells played a pronounced role in this immune response. In this work, we evaluated SBV nucleoprotein as a subunit vaccine, with or without a molecular adjuvant (i.e. Saponin). Saponin has been previously shown to induce a strong T-cell-based adjuvant response, including the stimulation of CD8 + T-cells^25^ through cross-presentation by dendritic cells through MHC class I, thus providing a means to stimulate cytotoxic T-cells^28^; and we hypothesized that Saponin might be able to help stimulate the immune response that we observed during our DNA vaccine studies. As a control, GFP was used, since: (a) it could also be readily expressed in bacteria, and; (b) it had a similar molecular weight to SBV-N.

Prior to virulent SBV challenge, we measured the presence of broadly virus-reactive antibodies from the serum of all vaccinated animals. Although mice in the SBV-N group were able to induce a high-level of broadly virus-reactive antibodies, the SBV-N + Saponin group was able to induce a higher level of SBV-antibody titers. IgG isotyping of the vaccinated mice also showed that in SBV-N (with or without Saponin), generated primarily IgG1 immunoglobulins; these results would be consistent with a Th1 response. Furthermore, the contribution of Saponin in increasing the immunogenicity of SBV-N was confirmed through cell-proliferation assays, where the SBV-N + Saponin group displayed greater CD4 + and CD8 + T-cell proliferation relative to the SBV-N group (and the GFP negative control groups). However, due to the small sample size (n = 3), the results were not able to reach statistical significance. Other factors may have also contributed to this which include the lack of optimized in vitro cell proliferation conditions, as well as the possibility that the stimulated T-cells were located outside the spleen (i.e. PBMCs, pancreas or other organs). Qualitatively, both the SBV-N and SBV-N + Saponin were capable of inducing IFN- γ secretion; however, as in the case of the cell-proliferation assay, the small sample size prevented the data from being statistically significant.

Following SBV challenge, we looked for clinical signs normally associated with SBV infection in IFNAR^-/-^ mice (i.e. weight loss). In our previous study, we found that unprotected mice showed a decrease in weight of approximately 5% within 7 days when challenged with SBV 80/11-4 strain. However, when using the more virulent SBV BH619/12 strain, no statistically significant changes in weight loss were observed in any groups.

These findings were further confirmed when viremia was measured following SBV challenge. Mice in the SBV-N + Saponin group showed significantly less presence of SBV, with an undetectable presence of the virus at days 10 and 13. Therefore, the SBV-Nucleoprotein, in combination with a veterinary grade Saponin, can be an efficient vaccine candidate against SBV infection. These findings are consistent with results from our previous study, where DNA vaccines were designed based on ORFs of the SBV genome. While we found two candidates (SBV-N and SBV-Gc ecto-1), we decided to pursue using SBV-N as a subunit vaccine, based on its ability to be readily expressed in *E.* *coli*, where large-scale production can be used to produce a more economically practical vaccine candidate.

Currently, there are licensed vaccines against SBV. These vaccines are inactivated, and have been shown effective in reducing the incidence of the disease. However, despite being made available within two years of the first SBV outbreak (in 2011), the lack of vaccine demand has led to a decrease in production, with two manufacturers temporarily halting SBV vaccine production^15,16^, with production only resuming following the 2016 outbreak. One possible reason for the lack of demand involves the cost-benefits associated with the vaccine^15^. Therefore, one way to remedy this problem would be to provide a cheaper, yet effective alternative to current SBV vaccines. Production of bacterially-expressed SBV-N would be significantly cheaper than the large-scale production of purified, inactivated SBV vaccines. Even when the cost of veterinary-grade (i.e. partially purified) Saponin is factored in, the production cost/dose would be significantly less that all current SBV vaccine candidates. We have provided a tentative cost-analysis of the vaccine that we produced in our laboratory (see Supplementary Table S1), and we have determined that for our experiments in mice, each experimental dose costed approximately 0.07 €.

Aside from providing a novel, more practical vaccine candidate against SBV infection, the results from this study may also have applications to other emerging bunyaviruses. It has been previously shown that the nucleoprotein from Rift Valley fever virus (RVFV) can confer partial protection against viral challenge; and that cDNA vaccination work demonstrated that a cDNA encoding for ubiquitinated RVFV nucleoprotein can confer nearly complete protection against virus challenge^29,30^. It is also worth noting that recombinant Crimean Congo Hemorrhagic fever virus (CCHFV) nucleoprotein was previously shown to have multiple CD8 + T-cell epitopes^31^ and that mice surviving Crimean Congo Hemorrhagic fever virus (CCHFV) challenge, were found to have CD8 + T-cells that could secrete IFN-γ years after infection (by ELISpot in response to peptide stimulation), thereby confirming the role that these cells have in the host immune response.

Based on our results with bacterially-expressed SBV nucleoprotein, we hypothesize that other recombinant bunyaviral nucleoproteins may have immunoprotective properties, when administered with an appropriate adjuvant. However, to definitively evaluate SBV nucleoprotein’s ability to confer protection against viral challenge and to validate the efficacy of SBV nucleoprotein when co-administered with Saponin further vaccination studies are required using large ruminant animal models.

## Materials and methods

### Viruses and mice

The Schmallenberg virus strain BH619/12 (7th culture passage) was provided by the Friedrich-Loeffler-Institute (FLI) through the European Virus Archive (EVA), and dilutions of the virus stock were used directly for all subsequent mouse experiments. The mice used in the vaccination experiments were A129 IFNAR α/β^-/-^ (B & K Universal Ltd, UK). All animal experiments using SBV were performed at the BSL3 animal facilities of NEIKER Institute (Derio, Spain) or the BSL3 + animal facilities at CISA/INIA (Madrid, Spain), with all proposed experiments (including containment vaccination, viral challenge and euthanasia) adhering to the ethical guidelines for animal care and experimentation and having received institutional approval (see below).

### Generation, bacterial expression and purification of SBV subunit vaccine candidates

GFP was expressed using bacterial expression vector pET-28M-SUMO3-GFP (obtained from the Protein Expression Facility at the European Molecular Biology Laboratory). This construct contains SUMO3, to promote solubility and correct protein folding of native, non-denatured proteins, as well as a histidine-tag for subsequent purification^32^. For SBV nucleoprotein expression, the GFP was excised from the aforementioned vector, and the cDNA of SBV nucleoprotein (Genbank accession number H2AM13) was ligated into the vector through the *AgeI/XhoI* restriction sites.

Subunit vaccine candidates were expressed in *E.coli* BL21, with colonies being selected on LB-Kanamycin plates at 37 °C. Individual colonies were then cultured in LB broth with Kanamycin (50 µg/mL), and induced with 1 mM IPTG for 12 h at 30 °C. Each clarified cell lysate in non-denaturating and non-reducing conditions [1X phosphate-buffered saline (PBS) buffer with EDTA-free protease inhibitor cocktail] was incubated with Ni-NTA resin (ThermoFischer) in batch mode at 4 °C for two hours. Then protein-bound resin was eluted with 1X PBS buffer supplemented with 500 mM imidazole (Sigma-Aldrich). Eluted proteins were then further purified through an additional step of size-exclusion gel-filtration chromatography using a HiLoad 16/60 Superdex 200 pg column (GE Healthcare) pre-equilibrated in 1 X PBS for the SBV-N and a Superdex 16/60 75 pg column (GE Healthcare) for the GFP.

While GFP eluted as a single monomeric peak corresponding to its expected molecular weight (MW 26 kDa) (Fig. S3a), SBV-N eluted with multiple peaks, consistent with the multimerization of native protein as previously described^22^ with the largest fraction being localized to an elution volume of less than 80 mL (Fig. S3b). The expression and purity of each construct was evaluated by SDS-PAGE and Coomassie blue staining (Fig. S3) and their identity confirmed by mass-spectroscopy and peptide identification (*data not shown*).

### Immunization and SBV viral challenge

Six groups of adult IFNAR^-/-^ mice (with 4–6 mice per group) were inoculated with subunit candidates twice subcutaneously at two week intervals. All injected solutions were in a final volume of 100 µl. The six groups were as follows: (1) 50 µg SBV nucleoprotein; (2) 50 µg SBV nucleoprotein + 14 µg of Quil-A Saponin (InvivoGen, USA), (3) 50 µg GFP ; (4) 50 µg GFP + 14 µg of Quil-A Saponin,; (5) 14 µg of Quil-A Saponin, and; (6) 100 µl of PBS. Two weeks after the final vaccination, all mice were challenged with an intraperitoneal dose of 2 × 10^5.^^8^ TCID_50_ SBV (strain BH619/12-7), resuspended in 200 μl of DMEM. The changes in weights (as well as all hereafter generated data) were analyzed for statistical significance using a one-way ANOVA test (see below).

### Serological detection of broadly virus-reactive IgG

Broadly virus-reactive IgG was quantified using ELISA as previously reported^18^, with minor modifications. Briefly, serum from each mouse was obtained from blood collected one week after the final vaccination. ELISA plates were coated with enriched, heat-inactivated SBV, purified as described^18^. Then, the sera, heat-inactivated at 56 °C for 1 h, was added starting at a 1:1 × 10^3^ dilution and serially diluted to 1:2048 × 10^3^. The plates were incubated for 1 h at 37 °C. The plates were washed 3 times with PBS/0.1% Tween 20 and incubated with HRP-conjugated anti-mouse IgG (Sigma, USA). Following another three washes with PBS / 0.1% Tween, the plates were developed with 1-Step Ultra TMB substrate solution (Fisher Scientific, USA) for 30 min, then stopped with one volume of 2 M sulfuric acid and the absorbance measured at a 450 nm.

For IgG isotyping, ELISA plates were coated for 2 h with 5 µg/mL previously purified SBV nucleoprotein at room temperature and blocked with PBS/0.1% Tween 20 2% BSA overnight at 4 °C. Heat inactivated sera samples harvested two weeks after the last vaccination from mice belonging to the groups 1, 2 and 6 were added on duplicates serially diluted at 1:10 and 1:100. After 1 h incubation at 20 °C the plates were washed 4 times with PBS / 0.1% Tween 20 and incubated with HRP-conjugated secondary goat anti-mouse IgG1, IgG2a and IgG2b (Fischer Scientific, USA). Following another 4 washes with PBS/0.1% Tween and one wash with PBS, the plates were developed with 1-Step Ultra TMB substrate solution (Fisher Scientific, USA) for 30 min, the reaction was stopped with one volume of 2 M sulfuric acid and the absorbance measured at a 450 nm.

### Virus neutralization tests (VNT)

VNT experiments against SBV infection were performed as described elsewhere^19^. Briefly, sera collected from vaccinated mice were serially diluted, and incubated with 10^4^ TCID_50_ of SBV. The mixtures were added to BHK-21 cells in a 96-well plate, and monitored for cytopathogenic effect (CPE) after 4 days. All sera were tested in triplicates. In order to validate our VNT assays, control sera from both SBV-convalescing sheep, as well as uninfected sheep were used as positive and negative controls, respectively (with both sera being a kind gift from Dr. M. Beer (Friedrich-Loeffler-Institut, Greifswald, Germany).

### Detection and quantification of viremia by real time RT-qPCR

During the SBV viral challenge 100 µl of blood was collected at 3, 6, 10 and 13 days post-infection (dpi). RNA was extracted using the Paramagnetic Beads RNA extraction kit (Life River, Shanghai ZJ Biotech, China) according to the manufacturer’s instructions, and eluted in 50 µl. The presence of SBV RNA was detected using the SBV dtec-RT-qPCR kit (Genetic PCR Solutions, Elche, Spain) using 5 µl of the extracted RNA samples. Real-time PCR was performed on an Agilent 3005 P Real-time PCR system, using the FAM channel for quantification, and the HEX channel to measure the internal controls provided by the manufacturer. The thermal profile used was as follows: 50 °C for 10 min, 95 °C for 60 s, followed by 40 cycles of the following: 95 °C for 10 s and 60 °C for 60 s.

### Cell proliferation assay

To determine the role of cellular immunity in response to SBV vaccination in IFNAR^-/-^ mice, cell proliferation assays using mouse splenocytes were performed. Mice were vaccinated using an identical schedule, as specified above, and included the following groups (with 3 mice per group): (1) 50 µg SBV nucleoprotein; (2) 50 µg SBV nucleoprotein + 14 µg of Quil-A Saponin; (3) 50 µg GFP, and; (4) 50 µg GFP + 14 µg of Quil-A Saponin. One week following the final vaccination, all of the mice were euthanized, and the splenocytes were isolated in a manner previously described^33^. Briefly, isolated splenocytes were adjusted to a concentration of 10^6^ cells/mL and stained with 5 µM CellTrace CFSE cell proliferation kit (Molecular Probes, USA) in DMSO, according to the manufacturer’s instructions. Cells were then resuspended to a final concentration of 1 × 10^6^ cells/mL in RPMI 1640 medium supplemented with 10% fetal calf serum, and seeded in a 96-well plates pre-coated with 500 ng/well of anti-CD3e (BD Biosciences, USA) in triplicate in the presence of highly enriched heat-inactivated SBV^18^. Phytohaemagglutinin (PHA) was used as a positive control at 5 µg/mL, whereas the negative control used was medium without antigen. The plates were incubated for 3 days at 37 °C at 5% CO_2_. After incubation, the cells were washed, immunostained for CD8 and CD4 (PE-anti-CD8a and APC-anti-CD4, respectively-BD Biosciences) and fixed. Data were acquired using FACScalibur (BD Bioscience, USA) and analyzed using FlowJo v10.6.1 software. This software enables for the evaluation of the green fluorescence of the CFSE, which in turn, was used to determine the proportion of dividing cells within each CD4 + and CD8 + cell population.

### ELISA assays for the detection of secreted IFN-γ

Splenocytes from the cellular proliferation assay also served to quantify secreted IFN-γ. The stimulation conditions (i.e. inactivated SBV) used were identical to the conditions used for the cell proliferation assays. Briefly, 96-well High Binding Costar 3590 were coated with 2 µg/ml of anti- IFN- γ capture antibody AG-18/RA-6A2 (BD Pharmingen). Following 1hour incubation at 37 °C, the wells were washed two times with PBS/0.05% Tween 20 and blocked with PBS/0.05% Tween/0.1% BSA for 1 h at 37 °C. 50 ul of supernatant was added to each well and incubated for 1 h at 37 °C. The plates were then washed with PBS / 0.05% Tween and incubated with 1 mg/ml of anti- IFN- γ biotinylated mAb R46A2 (BD Pharmingen) for 1 h at 37 °C. Afterwards, plates were washed with PBS / 0.05% Tween and 50 µl of peroxidase-labeled streptavidin at a 1/500 dilution in PBS added to each well and incubated at 37 °C for 1 h. After 1 h at 37 °C plates were washed, and the TMB substrate (Sigma-Aldrich) was added for 10 min, followed by one volume of stopping solution (0.5 M sulfuric acid). Optical densities were measured at 450 nm.

### Statistical analysis

The following sample sizes of adult IFNAR^-/-^ mice were selected in compliance with the three Rs (Replacement, Reduction and Refinement) principle in animal experimentation^34^ and previous studies^18^: (i) SBV-N and SBV-N + Saponin (n = 6 each group); (ii) GFP and GFP + Saponin (n = 4 each group); (iii) Saponin (n = 4); and (iv) PBS (n = 5). The one-way analysis of variance (ANOVA) was used to assess whether there are any statistically significant differences between the resulting means of the different experiments when more than two groups were considered. When analysis was performed across two groups only [*eg*. SBV-N *versus* PBS (control)] then the unpaired t-test with Welch’s correction was used to take into account the unequal variance and possible sample sizes. To this end we used GraphPad PRISM version 8.4.2.

### Ethics statement

The study was approved by the *Diputacíon Foral de Bizkaia* 12/2018 for experiments carried out at the NEIKER-Basque Institute for Agricultural Research and Development and by the *Comunidad de Madrid* permit PROEX 108/15 for research performed at the Animal Health Research Center (INIA-CISA). All experiments were monitored by staff veterinarians and animals that exhibited severe signs of morbidity were euthanized by cervical dislocation.

## Supplementary information

Supplementary Information.
