# Supplementary material for: A novel Schmallenberg virus subunit vaccine candidate protects IFNAR-/- mice against virulent SBV challenge
Source: Sci Rep. 2020 Nov 23;10:18725. doi: 10.1038/s41598-020-73424-2 (PMC7684302; doi:10.1038/s41598-020-73424-2)
Supplement: Supplementary file 1 — Supplementary Information. [file 41598_2020_73424_MOESM1_ESM.docx]

**SUPPLEMENTARY INFORMATION**

**A novel Schmallenberg virus subunit vaccine candidate protects IFNAR^-/-^ mice against virulent SBV challenge**

Hani Boshra^1,†,*^, Gema Lorenzo^2^, Diego Charro^1^, Sandra Moreno^2^, Gabriel Soares Guerra^1^, Isbene Sanchez^3^, Joseba M. Garrido^4^, Marivi Geijo^4^, Alejandro Brun^2^, Nicola GA Abrescia^1,5,6,*^

^1^Center for Cooperative Research in Biosciences (CIC bioGUNE), Basque Research and Technology Alliance (BRTA), Bizkaia Technology Park, 48160 Derio, Spain.

^2^Animal Health Research Center (INIA-CISA), Valdeolmos, 28130 Madrid, Spain.

^3^Vacunek SL, Bizkaia Technology Park, 48160 Derio, Spain.

^4^Animal Health Department, NEIKER-Basque Institute for Agricultural Research and Development, Derio, Bizkaia, Spain

^5^IKERBASQUE, Basque Foundation for Science, 48013 Bilbao, Spain.

^6^Centro de Investigación Biomédica en Red de Enfermedades Hepáticas y Digestivas (CIBERehd), Instituto de Salud Carlos III, Madrid, Spain

^†^Current address: Department of Pathology, Fundamental and Applied Research for Animals & Health (FARAH), University of Liège, Faculty of Veterinary Medicine, Bât B43, avenue de Cureghem 6, 4000 Liège, Belgium, Tél: +32 4 3664075.

*Corresponding authors. *E-mail address:* [hboshra@uliege.be](mailto:hboshra@uliege.be), [nabrescia@cicbiogune.es](mailto:nabrescia@cicbiogune.es)


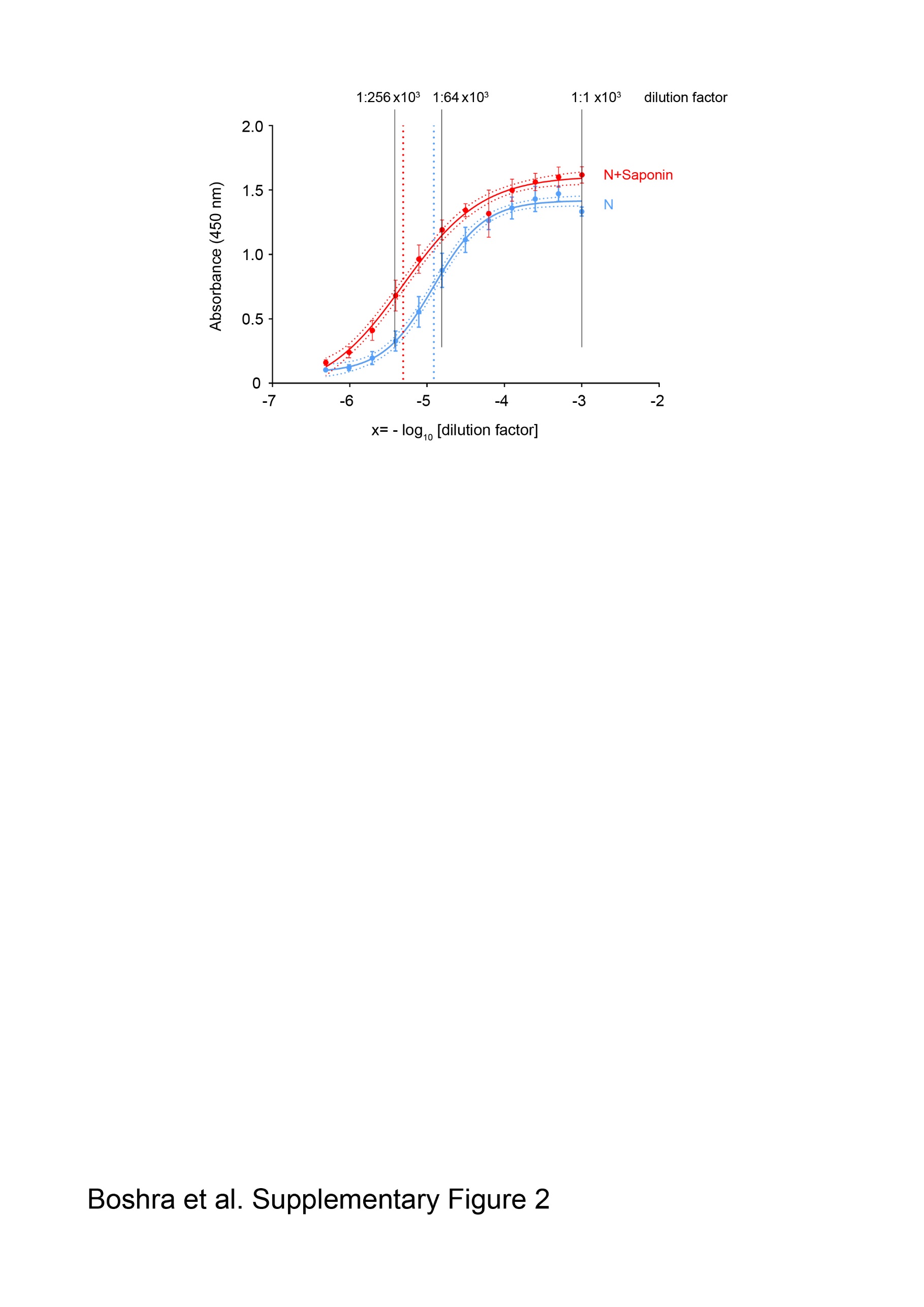


**Fig. S1**. **Comparison of half maximal effective concentration (EC_50_) curve response of anti-N with and without Saponin**

The curves and EC50 values were obtained by interpolating the data of the ELISA sera for N and N+Saponin (Fig. 1) using a four parameter logistic sigmoidal curve in GraphPad. In red the curve corresponding to the antibodies generated by vaccination with N+Saponin at different dilutions whereas in blue the curve of the antibodies generated by vaccination with only the N protein. The dotted lines represent the concentration of the corresponding antibodies where 50% of their maximal effect is observed; note that in this case less amounts of the N+Saponin serum than that of N alone is needed to achieve 50% OD values in the ELISA (Fig. 1); the error bars indicate the standard error of the mean.


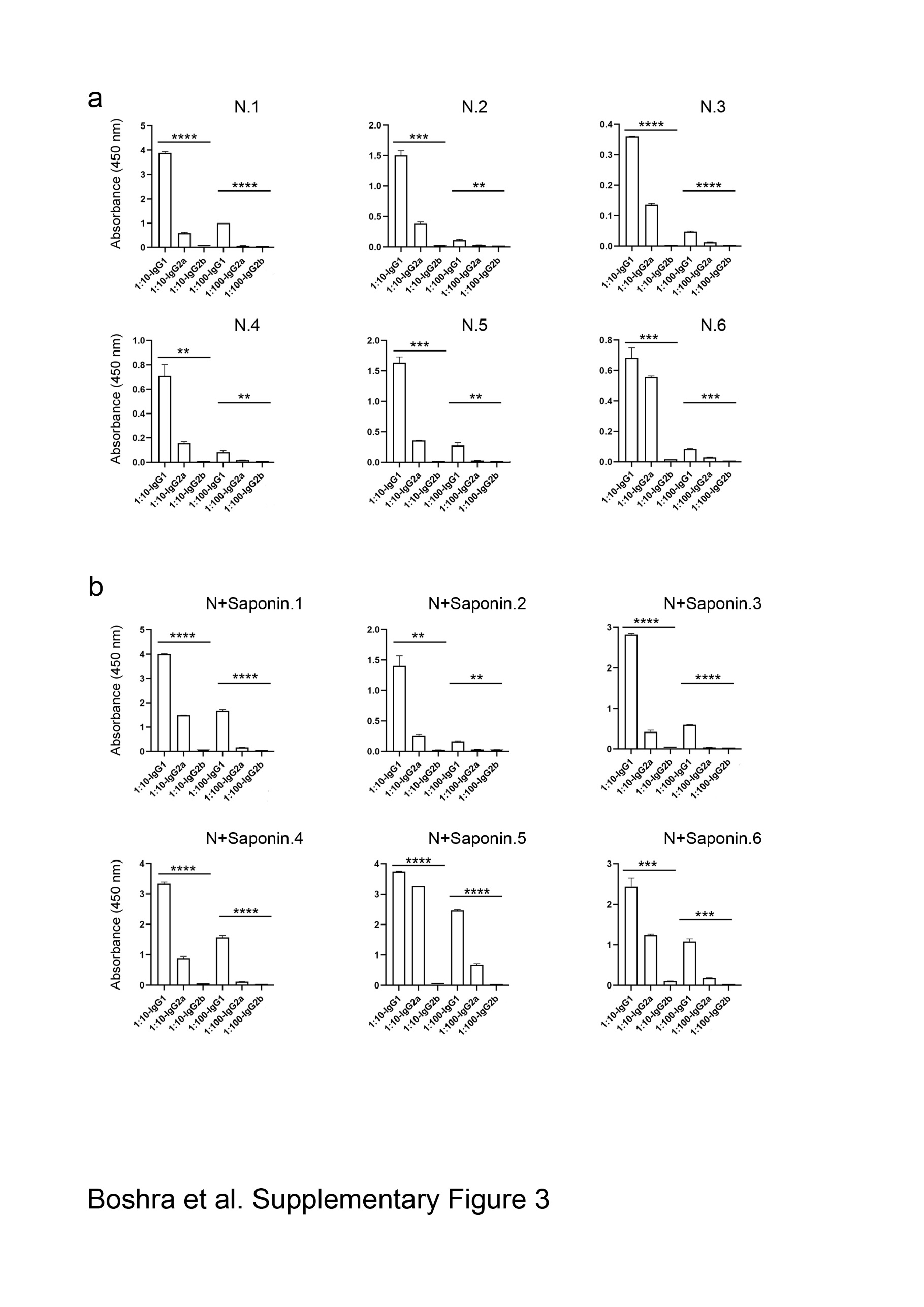


**Fig. S2**. **ELISA IgG isotyping of the antibody response of mice vaccinated with N and N+Saponin**

(**a**) Group of mice (n = 6) vaccinated using the subunit protein N. Two dilutions of sera (1:10 and 1:100) were used and in duplicate (error bar represents the SD between the duplicate OD readouts) whereas the (*) indicates the statistical significance across the three groups IgG1, IgG2a and IgG2b at the two dilutions. (**b**) As **a** but using vaccinated mice with N+Saponin.


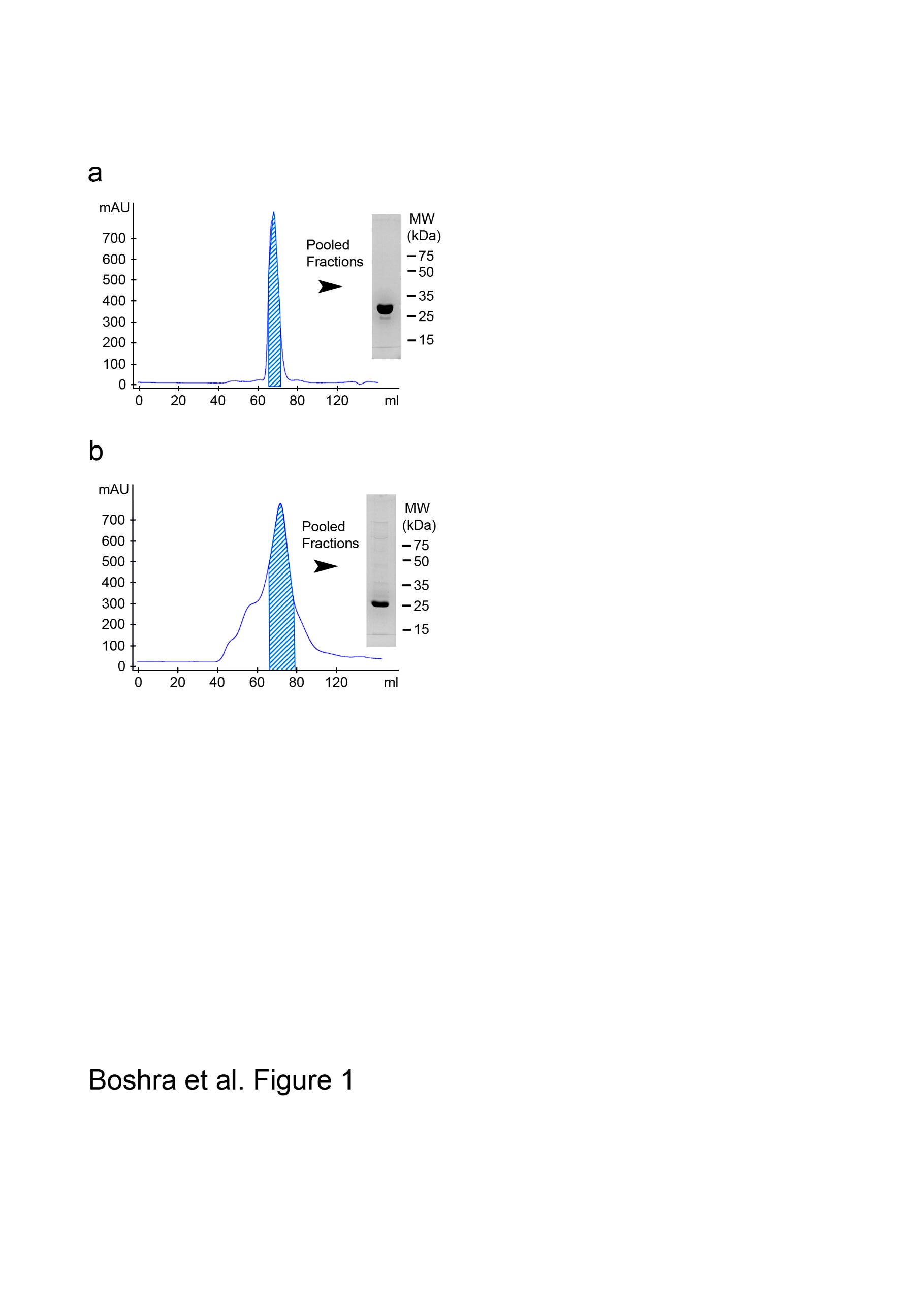


**Fig. S3**. ***In vitro* expression of recombinantly expressed SBV nucleoprotein (SBV-N) and green fluorescent protein (GFP), expressed in *E.coli* BL21**

(**a**) Purified GFP passed through a Superdex 16/60 75 pg gel filtration column. (**b**) Purified SBV-N passed through a Superdex 16/60 200 gel filtration column. Fractions that were to be used for subsequent vaccination studies are indicated by the diagonal line below each curve of the chromatogram. The pooled fractions were also visualized for purity by 10% SDS-PAGE and Coomassie blue staining (**a**, **b** right).

**Supplementary Table S1.**

**Estimated breakdown cost of consumables for preparing SBV-N + Saponin on an analytical scale for mouse experimentation**

| Item | Cost |
| --- | --- |
| Bacterially expressed SBV-N protein—Keeping in mind that 1L of bacterial culture is sufficient to produce up to 5 mg of protein—(LB+Kanamycin+IPTG+ 1L deionized water) | 5 € (for 5 mg of protein) |
| Purification of SBV-N protein on 2 mL of Ni-NTA resin (400 € / 25mL, which can be re-used 10 times). Including the price of PBS and Imidazole | 1 € (for 5 mg of bound protein) |
| Purification and Size exclusion gel chromatography—Price of PBS (used from 10X stock and diluted with deionized water) | 1 € per liter (which is sufficient to purify 5 mg of recombinant protein) |
| Veterinary-grade Saponin—179 € / 1 gram. Considering that each mouse received 14 micrograms, the amount per mouse (per dose) | 0,002 € |

Cost to purify 5 mg of SBV-N + 14 µg of veterinary-grade Saponin 7 €

Cost per dose (in mice) 0.07 €
